# Computational and Experimental Approaches Exploring the Role of Hesperetin in Improving Autophagy in Rat Diabetic Retinopathy

**DOI:** 10.3390/biomedicines12030552

**Published:** 2024-03-01

**Authors:** Reem Alshaman, Eman Kolieb, Rehab M. El-Sayed, Sahar Galal Gouda, Abdullah Alattar, Sawsan A. Zaitone, Asmaa K. K. Abdelmaogood, Lamiaa M. Elabbasy, Amira H. Eltrawy, Fai Yahya Sayd, Hatem I. Mokhtar, Esam Ghanem Abu El Wafa, Esam Sayed Ahmed, Dong Liang, Dina A. Ali

**Affiliations:** 1 Department of Pharmacology and Toxicology, Faculty of Pharmacy, University of Tabuk, Tabuk 47512, Saudi Arabia; 2 Physiology Department, Faculty of Medicine, Suez Canal University, Ismailia 41522, Egypt; 3 Department of Pharmacology and Toxicology, Faculty of Pharmacy, Sinai University, El-Arish, North Sinai 45511, Egypt; 4 Department of Histology and Cell Biology, Faculty of Medicine, Suez Canal University, Ismailia 41522, Egypt; 5 Department of Pharmacology and Toxicology, Faculty of Pharmacy, Suez Canal University, Ismailia 41522, Egypt; 6 Department of Clinical Pathology, Faculty of Medicine, Suez Canal University, Ismailia 41522, Egypt; 7 Department of Medical Biochemistry & Molecular Biology, Faculty of Medicine, Mansoura University, Mansoura 35516, Egypt; 8 Department of Basic Medical Sciences, College of Medicine, Almaarefa University, Diriyah, Riyadh 13713, Saudi Arabia; 9 Department of Anatomy, Faculty of Medicine, University of Tabuk, Tabuk 47512, Saudi Arabia; 10 Department of Anatomy and Embryology, Faculty of Medicine, Alexandria University, Alexandria 21526, Egypt; 11 PharmD Program, Faculty of Pharmacy, University of Tabuk, Tabuk 71491, Saudi Arabia; 12 Department of Pharmaceutical Chemistry, Faculty of Pharmacy, Sinai University-Kantara Branch, Ismailia 41636, Egypt; 13 Department of Ophthalmology, Al-Azher Asyut Faculty of Medicine for Men, Asyut 71524, Egypt; 14 Department of Pharmaceutical & Environmental Health Sciences, College of Pharmacy and Health Sciences, Texas Southern University, Houston, TX 77004, USA; 15 Clinical Pharmacology Department, Faculty of Medicine, Suez Canal University, Ismailia 41522, Egypt; 16 Center of Excellence in Molecular and Cellular Medicine, Suez Canal University, Ismailia 41522, Egypt

**Keywords:** autophagy, beclin 1, computational approach, diabetic retinopathy, hesperetin, rat

## Abstract

Diabetic retinopathy (DR) is a debilitating diabetic disorder of the retinal microvasculature and the main cause of avoidable blindness in old people. Hesperetin is a plant flavanone largely abundant in citrus species with neuroprotective properties in animal models. This study aimed to explore the neuroprotective and autophagy-enhancing effect of hesperetin in rats with DR. Twenty-four male rats were utilized and allocated to groups: (i) the vehicle group, (ii) DR group and (iii–iv) the DR + hesperetin (50 and 100 mg/kg) groups. Treatment with hesperetin continued for 6 weeks. After the rats were euthanized, their eyes were dissected to detect the biochemical and histological changes in the retinas. Quantification of autophagy markers, beclin 1/LC3/p62, and inflammation markers was performed. Histopathologic changes were investigated after staining with hematoxylin and eosin and periodic acid–Schiff (PAS). Results demonstrated that hesperetin decreased the PAS staining in diabetic rats and attenuated histopathological changes and restored retinal organization and thickness of layers in hematoxylin and eosin staining. Moreover, hesperetin reduced the level of mRNA expression for *TNF-α* (4.9-fold), *IL-1β* (4.15-fold), *IL-6* (4.6-fold) and *NFκB* (5.2-fold), as well as the protein level. This was accompanied by induction of autophagy proteins, beclin 1 and LC3-II. Our results afford evidence that hesperetin is effective in alleviating the pathology of DR via suppressing the inflammatory burden and induction of autophagy. After extensive clinical examinations, hesperetin may prove to be a useful option for treatment of DR.

## 1. Introduction

The most prevalent microvascular consequence of diabetes is diabetic retinopathy (DR) [1], and if left untreated, it can lead to vision loss and in severe cases blindness [2]. Disrupting pericyte–endothelial cell interactions worsened diabetes-induced microvascular dysfunction [3]. Early DR was also associated with endothelial cell death, basement membrane thickening [4] and retinal apoptotic death [5,6,7]. Currently, a major factor in the pathophysiology of DR is the persistent, low-grade inflammation. The early alterations in diabetic retinal degeneration include the disruption of the blood–retina barrier [8], which allows blood to seep into the retina. This is largely due to the action of inflammatory cells, such as macrophages and microglia. Additionally, they play a significant part in the pathophysiology of the more severe stages of proliferative DR, which can result in vitreous hemorrhage, tractional retinal detachment, and neovascularization [9,10].

Autophagy, a key lysosomal mechanism, removes cytoplasmic organelles and long-lived proteins and serves as both a homeostatic and adaptive response to cell stress [11]. Data from the laboratory and clinic research reveal that autophagy can be modulated in DR animal models and diabetic humans [12,13].

Autophagy acts as a mediator between innate and adaptive immunity. Inadequate autophagy can lead to inflammatory reactions, cellular death, and inherited instability. Neurodegeneration and inflammation are all closely associated with autophagic dysfunction [14]. From a therapeutic standpoint, autophagy appears to be an attractive target mechanism due to its crucial function in eliminating the major toxic entity causing disease and consequently reducing sensitivity to pro-death insults [14]. Autophagy is thought to play a role in DR, but how exactly it does this is unknown. Here, we examine autophagy’s function in an experimental model of DR and investigate its promise as a target for novel treatment interventions.

Beclin 1 is crucial for localizing autophagy proteins to the preautophagosomal structure, playing an essential role in autophagy onset and serving as a marker for monitoring autophagy [15]. Autophagy includes converting LC3-I into LC3-II, with the LC3BII/LC3BI ratio commonly used as a marker for autophagosome formation [16]. P62 is the ubiquitin-binding protein linked to degradation of proteins [17]. During autophagy, P62 can bind to the cytoplasmic ubiquitinated proteins, then localizes with LC3-II on the autophagosome, and is finally degraded in autolysosomes. Hence, P62 degrades during the progression of autophagy, and is known to be expressed in an inverse correlation with autophagy [18].

Although anti-angiogenesis therapies have been shown to be effective, they are not yet of much benefit in the early stages of DR, leaving patients at danger of blindness [19]. Therefore, there is an urgent need for novel findings concerning pharmacological compounds that might effectively impede or halt the progression of DR at an early stage. The bioactive molecule hesperetin ((2S)-5,7-dihydroxy-2-(3-hydroxy-4-methoxyphenyl)-3,4-dihydro-2H-1-benzopyran-4-one, CAS Registry Number 520-33-2) is the aglycon of the flavonoid glycoside hesperidin, which is found in citrus fruits (family Rutaceae). Hesperetin is suggested to be the absorbed form of the parent flavonoid, hesperidin, after removal of its glycosidic sugar part by the intestinal flora microbial enzymes [20]. It has been shown to reduce capillary permeability and fragility, making it an important component of Chinese traditional medicine [21]. Models of neurodegeneration have demonstrated that hesperetin downregulates lipopolysaccharide-induced neuronal apoptosis and neuroinflammation [22,23]. Many chronic inflammatory conditions have been successfully prevented and treated with hesperetin [23,24,25]. Accordingly, Choi et al. documented that hesperetin decreased levels of inflammatory mediators like prostaglandin E2, nitric oxide, tumor necrosis factor alpha (TNF-α), and interleukin-6 (IL-6) in a concentration-dependent manner [20].

Furthermore, a recent study reported profound neuroprotective properties for hesperetin in neurodegenerative diseases [26]. Moreover, other recent evidence described that it has neuroprotective and antidiabetic effects [27]. Further, Yang et al. reviewed the potential therapeutic role of hesperetin in the prevention and mitigation of diabetes and its complications [28], but the mechanism is still to be investigated.

No previous studies have tested the stimulating effect on autophagy as a possible mechanism that may mediate the neuroprotective effect of hesperetin in DR. Hence, the current research was designed for investigating the neuroprotective effect of hesperetin by applying a computational approach and conducting an experimental DR rat model. We focused on the impact of hesperetin on diabetes-mediated inflammatory burden and defective autophagy.

## 2. Materials and Methods

### 2.1. Methodology of the Computational Approach to Investigate the Relation between the Target Proteins and DR

Using freely available databases, 3 terms were used in our search entry: pathways of neurodegeneration/diabetic retinopathy/autophagy. Targeted genes/proteins in all the three entries were retrieved for comparative purposes. The databases were STRING 12.0 database (https://string-db.org/) accessed on 10 December 2023 [29], Online Mendelian Inheritance in Man (OMIM) at https://omim.org/ accessed on 11 December 2023 [30], DisGeNET (v7.0) (v7.0) at http://www.disgenet.org/ accessed on 12 December 2023 [31], Therapeutic Target Database (TTD) at https://db.idrblab.net/ttd/ [32] accessed on 12 December 2023 and Kyoto Encyclopedia of Genes and Genomes (KEGG pathway database: https://www.genome.jp/kegg/pathway.html) accessed on 13 December 2023 [33].

Gene/protein lists from the previous entries were exported from all the datasets and combined with duplicate removal. This aimed at exploration of the shared putative targets using FunRich software version 3.1.3 (www.funrich.org) accessed on 16 December 2023, which was used to compare the lists and visualize the overlapping genes [34]. This list of the shared targets served later as the entry gene query for in-depth gene characteristic analysis and exploration of the enrichment utilizing ShinyGO 0.77 at bioinformatics.sdstate.edu/go, accessed on 18 December 2023, with *Homo sapiens* as species and cutoff set for FDR at 0.05 [35].

Furthermore, the list was investigated via the STRING 12.0 database to explore and visualize protein–protein interactions (PPIs) to allow the researchers to navigate through the shared proteins and make the best choice for the upstream experiments [36].

### 2.2. Animals

In the current study, 24 male Wistar albino rats with body weight of 150 to 172 g were used and housed in plastic cages in groups of three. Rats were allowed to acclimatize to the housing conditions for 8 days at constant temperature and free access to feed. Authors adhered to the ARRIVE criteria and the Guide for the Care and Use of Laboratory Animals by the National Research Council throughout the experiment. This study was permitted by the Research Ethics Committees at the Faculty of Pharmacy (202302RA10) and the Faculty of Medicine (#5274) at SCU.

### 2.3. Chemicals and Drugs

Streptozotocin (STZ, >99%, MWt = 265.2) powder and hesperetin were purchased from Sigma-Aldrich Company (St. Louis, MO, USA). STZ solution was prepared in 0.01 M citrate buffer with pH 4.4, while hesperetin was suspended in 1% carboxymethylcellulose (CMC) solution.

### 2.4. Experimental Model of Type 1 Diabetes Mellitus

**Figure 1:**
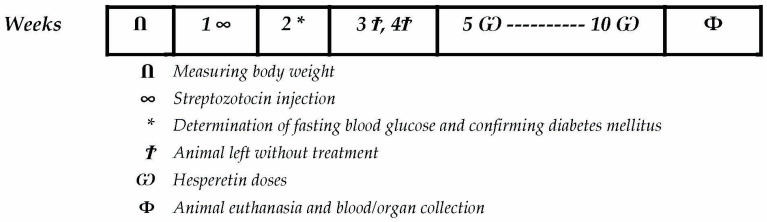
Diagrammatic presentation for the course of the animal experiment.

After 8 days’ acclimatization, six animals serving as the vehicle group were injected with an equal volume of citrate buffer. For the rest of the animals, diabetes mellitus was induced by 2 STZ injections (30 mg/kg) following a recent schedule [37,38] in fasted rats. Then, fasting blood glucose level was determined using a GlucoDr. glucometer (AllMedicus Co. limited, Gyeonggi-do, South Korea) to verify the induction of diabetes. Rats showing glucose levels exceeding 250 mg/dL were assigned to the experiment. Diabetic rats were left for 2 weeks until the development of DR pathology. Then, rats received daily doses (by oral gavage) of hesperetin (50 and 100 mg/kg) [39,40] or 1% CMC solution for 6 weeks (Figure 1).

### 2.5. Design of the Experiment

The animals were allocated into four groups (6 rats in each group).

- Vehicle group: normal rats received 2 injections (1 mL/kg) of citrate buffer parallel with STZ injections.
- DR group: diabetic rats received 2 injections (1 mL/kg) of STZ and served as a DR control group. Rats received oral doses of distilled water (2 mL/kg) parallel with hesperetin.
- DR + hesperetin 50 mg/kg group: diabetic rats received daily oral hesperetin (50 mg/kg), started 2 weeks after confirming diabetes and continued for 6 weeks [39].
- DR + hesperetin 100 mg/kg group: diabetic rats received daily oral hesperetin (100 mg/kg) started 2 weeks after confirming diabetes and continued for 6 weeks [40].

### 2.6. Blood Samples, Scarification and Retinal Dissection

Under the influence of ketamine (100 mg/kg, intraperitoneal) anesthesia, euthanasia was carried out on the rats by cervical dislocation. Following that, blood samples were collected from the retroorbital plexus and allowed to stand at room temperature for 25 min and centrifuged for 8 min at 1500× *g*. Serum samples were separated and kept at −80 °C for further investigating the target proteins. The left eyeballs were dissected, and the cleared-out eyeballs were fixed in Davidson’s solution for 48 h, then dehydrated in different grades of ethanol, and processed for preparation of 5 µm paraffin sections for histological and immunohistochemical assessment. The right eyeballs were processed to dissect the retinas, which were immediately frozen at −80 °C in separate containers. A portion of the retinal tissue was subjected to homogenization in phosphate-buffered saline, followed by 13 min of centrifugation at 2000× *g*. The resulting supernatants were then frozen and used later for ELISA assays. Other frozen portions were used for PCR assay and Western blotting.

### 2.7. Analysis of Serum Advanced Glycation End Products (AGEs)

Hyperglycemic accumulation of AGEs is implicated in the development of DR. Hence, serum AGE levels were quantified with a rat AGE enzyme-linked immunosorbent assay (ELISA) kit from My BioSource (MBS261131). The assay depends on a double-antibody sandwich technique for binding AGEs between anti-rat AGE monoclonal antibody and the detection of biotinylated polyclonal antibodies, followed by the addition of horseradish peroxidase (HRP) and avidin. The optical density of the color was measured at 450 nm after the addition of the HRP substrate. AGE serum concentrations were calculated by comparison with a standard curve of AGE serial dilutions prepared by the same kit.

### 2.8. Analysis of Retinal MDA, Inflammation Markers and Beclin 1

For the other assays, the retinal tissue homogenates were subjected to centrifugation at 1500× *g* and 4 °C for a duration of 23 min to eliminate solid particulate matter, then the supernatant aliquots were utilized for the detection of malondialdehyde (MDA, an oxidation marker), inflammatory cytokines and beclin 1.

MDA in retinal tissue homogenates were measured using a colorimetric kit from Biodiagnostics (Cairo, Egypt). The kit depends on the colorimetric measurement at 534 nm of the reaction product of thiobarbituric acid and lipid peroxidation products.

Kits for ELISA based on a double-antibody sandwich technique were applied for the assay of TNF-α (MBS2500421), IL-6 (MBS355410) and beclin 1 (MBS733192). The kits were purchased from MyBiosource Inc. Company (San Diego, CA 92195-3308: USA). Readings were assessed in comparison with measured standard curves.

On the other hand, a competitive immunosorbent ELISA technique was applied for NFκB determination using an NFκB (MBS722386) ELISA kit purchased from MyBiosource Inc. Company (San Diego, CA 92195-3308: USA). The assay is based in using polyclonal anti-NFκB antibody in competition with NFκB–HRP conjugate. Optical density was measured at 450 nm and calculation by comparison with a measured standard curve.

### 2.9. RNA Extraction and RT-PCR Analysis

From the tissue homogenate, extraction of total RNA was performed utilizing Trizol (Invitrogen, Waltham, MA, USA). A NanoDrop ND-1000 spectrophotometer was used for assessment of RNA concentrations and purity. Complementary DNA (cDNA) was synthetized from one microgram of RNA utilizing a Applied Biosystems high-capacity cDNA reverse-transcription kit (Waltham, MA, USA). We conducted the real time PCR using SYBR Green Master Kit (Fermentas, Waltham, MA, USA) with the aid of Applied Biosystems software version 3.1 (StepOneTM, Waltham, MA, USA). Twenty microliters were utilized for the quantitative real-time PCR (qRT-PCR). In order to normalize expression of each gene, glyceraldehyde-3-phosphate dehydrogenase (GAPDH) was utilized as a housekeeping control. The comparative Ct method (2^−∆∆Ct^) was employed for calculation of the fold changes in gene expression [41]. Calculation of these Ct values was undertaken by Step-One PCR detection software. The Primer 3 software (version 4.1.0) was used to allocate the primer sets, and for determining the specificity of these sets, the Primer BLAST program was employed (https://www.ncbi.nlm.nih.gov/tools/primer-blast/) accessed on 10 November 2023. The list of primer sets is shown in Table 1.

### 2.10. Assessment of beclin 1, LC3-II and p62 Proteins by Western Blot Analysis

A sample of each frozen retina was homogenized in RIPA lysis and extraction buffer (Thermo Fisher, Waltham, MA, USA) supplemented with protease and phosphatase inhibitor cocktails (ThermoFisher, USA) by first sonicating for 20 s and then centrifuging for 15 min at 1200× *g* at 4 °C. After being collected in fresh tubes, the supernatant was kept on ice. A 10% solution of sodium dodecyl sulfate polyacrylamide gel was loaded with equal volumes of the tissue homogenates’ protein mixtures (5 µg) and a prestained protein molecular weight marker (161–0305, Bio-Rad, Hercules, CA, USA). After electrophoretic separation of proteins on a gel, they were transferred to a 0.22 mm nitrocellulose membrane. Following a washing and an hour of incubation in 5% Bio-Rad nonfat dry milk, the membrane was blocked and kept for incubation with the designated primary antibody solutions overnight: anti-beclin 1 (1:500, PA1-16857, Thermo Fisher, USA), anti-LC3 (1:2500, PA5-22990, Thermo Fisher, USA), anti-p62 (1:1000, PA5-20839, Thermo Fisher, USA) and β-actin (1:5000, MA1-140, Thermo Fisher, USA) at 4 °C with mild agitation. Then, we washed the blots and added secondary antibodies labeled with horseradish peroxidase (ab205719, Abcam, Cambridge, UK). We identified the bound proteins using the commercial reagent ECLTM Plus Western Blotting Detection System kit (Amersham BioSciencies, Buckinghamshire, UK). ImageJ version 1.54 (NIH, Stapleton, NY, USA) software was utilized to conduct the densitometric analysis [42].

### 2.11. Immunohistochemistry for Beclin 1 in Retinal Sections

For beclin 1 immunostaining, rabbit polyclonal primary antibody (beclin 1 rabbit pAb, dilution 1:100, ABclonal, Woburn , MA, USA, Catalog No. A7353) was used. We collected 5 μm thickness sections from the paraffin blocks on positively charged glass slides. After that, sections were dried by heating to 60 °C overnight in an oven to achieve adequate adhesion. Following deparaffinization and rehydration, antigen retrieval was conducted by irradiating sections in a microwave in 0.01 M citrate buffer with pH 6.0. Then, they were positioned in a humid chamber and incubated in peroxidase/AP blocker for 5 min followed by incubation in beclin 1 primary antibody for 60 min. The horseradish peroxidase label (HRP) was applied for 45 min and finally 3,3′-diaminobenzidine chromogen for 10 min (Mouse/Rabbit PolyDetector, Bio SB, Goleta, CA, USA). Counterstaining was performed with Meyer’s hematoxylin for 30 s. Further, negative controls were prepared using PBS solution instead of the specific primary antibody, while a cut section from the renal cortex was used as a positive control. For morphometric analysis, the mean color area percentage and the mean optical density (OD) [43] of beclin 1 immunostaining in retinal layers were measured utilizing ImageJ software (NIH, USA). The mean color area percentage is defined as the area fraction of specific color in an image or the area fraction of specific color relative to the background. Thirty captures were assessed per group.

### 2.12. Histopathological Staining by Hematoxylin and Eosin and Periodic Acid–Schiff Stains

Left eyeballs were fixed in Davidson’s solution for 48 h, then transferred to ascending grades of ethanol for dehydration, cleared in xylene, embedded in liquid paraffin, and then cut into 5 µm sections for histological, histochemical, and immunohistochemical staining. Histological hematoxylin and eosin (HE) staining was performed for outlining the general architecture of the retina, and the histochemical periodic acid–Schiff reaction (PAS), which is selective for basement membrane mucopolysaccharides, was performed. Quantitative parameters were assessed in the retinal sections as follows.

- The mean thickness (µm) of the rod and cone layer (RCL), inner nuclear layer (INL), outer nuclear layer (ONL), ganglion cell layer and nerve fiber layer (GCL + NFL), and total retinal thickness (×630 in HE-stained sections).
- The average count of ganglion cells/200 μm length of the GCL (×400 in HE-stained sections).
- The average area percentage of PAS-positive material in the basement membrane of retinal blood vessels (×630 in PAS-stained sections). The wall of retinal blood vessels is formed of flat endothelial cells (simple squamous endothelial cells) resting on basement membrane. Basement membrane has mucopolysaccharide, which is stained magenta (positive PAS reaction) with PAS stain. In DR, there is accumulation of mucopolysaccharides in the basement membrane. As such, there is increased thickness of basement membrane and increased area of PAS-positive reaction (magenta) in the wall of blood vessels.

Measurement was carried out with small captures of the retina in the area of the GCL and NFL, and the area percentage of magenta reaction (PAS-positive reaction) in the wall of blood vessels was assessed.

We performed quantitative assessment using ImageJ software (NIH, USA). Six high-power fields (HPFs) representing both central and peripheral retina per section using 3 sections per rat [44] were used. Briefly, we captured small areas of the retina that enclose blood vessels in the optic nerve fiber and GCLs. Then, each image was split into 3 channels—image, color, and then color deconvolution—producing 3 images of the original image (blue, magenta and green). After that, we applied functions—(1) image, (2) adjust and then (3) threshold—to the image to measure the fraction of magenta relative to the background.

### 2.13. Statistical Analysis and Data Presentation

We present the results of the experimental rat study as means ± standard deviation. To ensure that data were distributed normally, we applied the Kolmogorov–Smirnov test. For analyzing the statistical differences between groups, one-way ANOVA and Tukey’s test were performed at *p* value < 0.05 using version 9 of GraphPad Prism (San Diego, CA, USA). Every possible comparison between the study groups was made.

## 3. Results

### 3.1. Computational Study of Connection between DR and Autophagy

**Figure 2:**
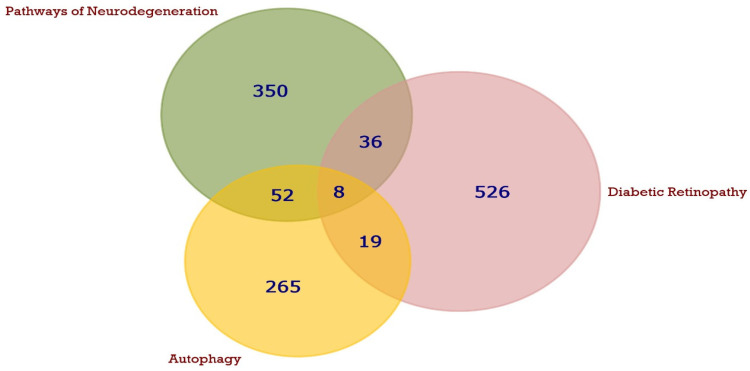
Venn diagrams show the shared putative targets (115 genes) between DR (big purple circle) and neurodegeneration pathways (green circle) and autophagy (small yellow circle) Analysis and building of figure were achieved utilizing version 3.1.3 of FunRich software.

Targeted gene/protein investigation using the abovementioned databases revealed the following. For neurodegeneration pathways, a list of 446 genes was retrieved. For DR, this mapped to 589 exclusive targets. For autophagy, 344 unique targets were revealed. FunRich software was used to map 115 intercalating genes/proteins shared between the three pathways in question (Figure 2).

**Figure 3:**
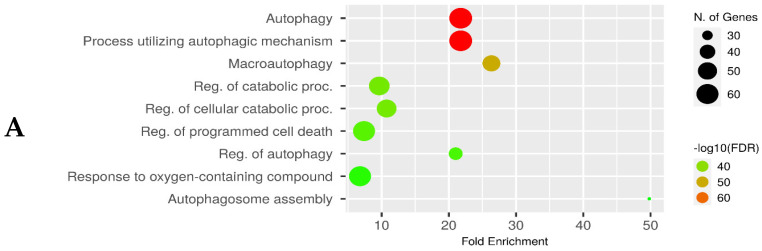

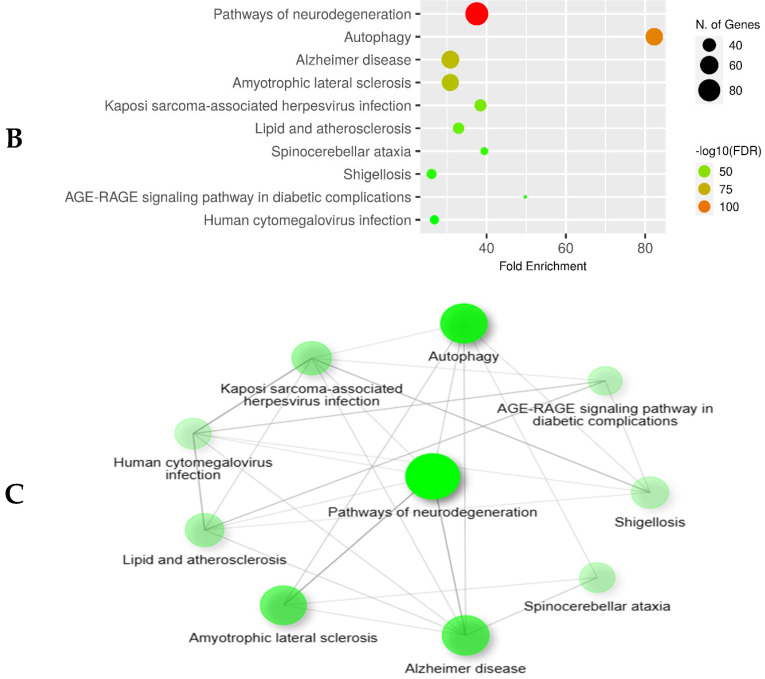
(**A**) Dot plot chart of top biological pathways enriched in the three proposed pathways. Bigger dots indicate higher significance of *p*-values. Chart was built utilizing ShinyGO 0.77 software. (**B**) Dot plot chart created for the major pathways from analysis by KEGG enrichment for the overlapping genes for exploring the pathways enriched in neurodegeneration, DR and autophagy, with the larger dots representing highly significant *p*-values. Chart was built by ShinyGO 0.77 software. (**C**) Illustrative network of the ten top pathways (sorted by FDR) shared among the three processes according to KEGG pathway database enrichment analysis. ShinyGO 0.77 software was used to produce the figure.

The analysis via ShinyGO 0.77a demonstrated that most of the overlapping genes were entangled in the biology of autophagy, apoptosis, and catabolism regulation together with biological response to oxidative stress (Figure 3A). Furthermore, KEGG enrichment analysis, via the software for the same gene set, was undertaken for exploring the enriched pathways in the three processes. Interestingly, the analysis highlighted that most of the genes were enriched in pathways as autophagy, neurodegeneration and lipid and atherosclerosis, enhancing the concept of the intercalating connections among these processes (Figure 3B). Further analysis of these top pathways was undertaken and revealed that they are interconnected and intercommunicating (Figure 3C).

**Figure 4:**
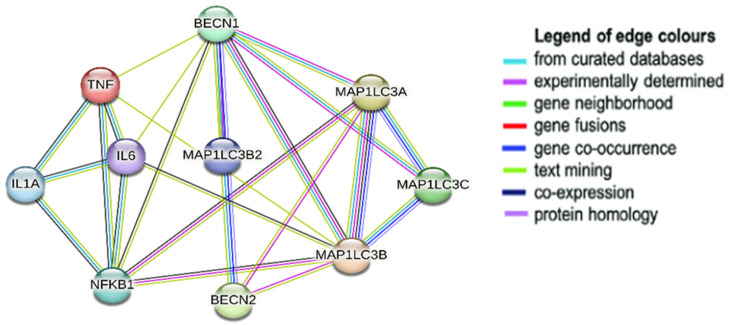
The candidate genes/proteins chosen for the experiment from two clusters. PPI network analysis revealed both functional and physical connection between them, even with the confidence set to its highest as 0.900. Network and analysis were conducted using STRING v.12.0.

PPI analysis in STRING using an interaction score of 0.900 highest confidence revealed an undeniable interaction between the genes and proteins. To ease the choice of proteins, clustering via k-means clustering algorithms was selected to obtain 2 main clusters (Supplementary Figure S1) from which the individual proteins were chosen depending on availability and with respect to the review of literature. Beclin 1, LC3, NFκB, TNF, IL1 and IL6 were chosen as representatives from the 2 clusters and they all maintained a strong functional protein association (Figure 4).

### 3.2. Effect of Hesperetin on Fasting Blood Glucose, Serum AGEs and Retinal Inflammation/Autophagy Markers

**Figure 5:**
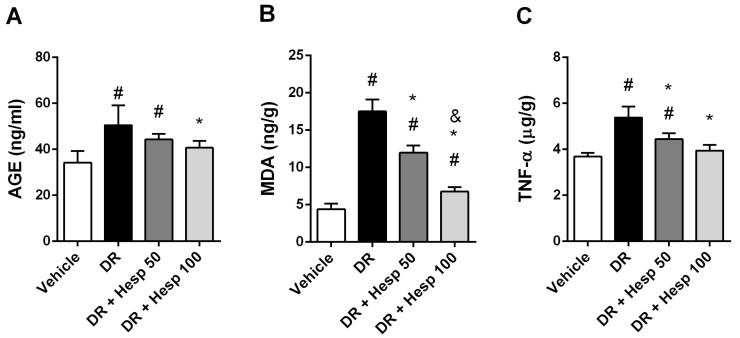

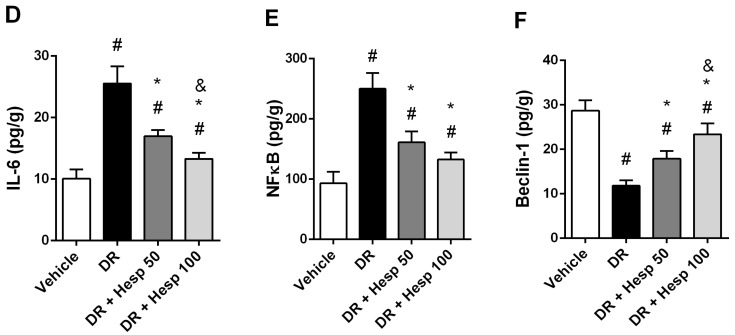
Effect of hesperetin on assays. (**A**) Serum AGE, (**B**) retinal malondialdehyde, (**C**) retinal TNF-α, (**D**) retinal IL6, (**E**) retinal NFκB and (**F**) retinal beclin 1 proteins. # Versus vehicle group, * versus DR group, and ^&^ versus DR + Hesp 50 mg/kg (*p* value < 0.05).

In the current study, fasting blood glucose level in the vehicle group was 102.67 ± 5.13 mg/dl versus 527 ± 68.84 in the DR group. DR + Hesp 50 or 100 mg/kg groups showed 433.67 ± 125.04 and 454.83 ± 78.79 mg/dl. DR control rats showed significant elevation in serum AGE and retinal MDA, TNF-α, IL-6, and NFκB levels. A significant decline in retinal beclin 1 level was observed. DR + Hesp 100 mg/kg showed a significant decline in serum AGE level versus the DR control group (Figure 5A).

Regarding the inflammatory parameters, retinal MDA, TNF-α, IL-6, and NFκB were significantly declined in DR + Hesp 50 or 100 mg/kg groups in comparison with DR control croup (Figure 5B–E). Retinal MDA and IL-6 in DR + Hesp 100 mg/kg group were significantly less than those of DR + 50 mg/kg group. Furthermore, retinal TNF-α levels in DR + 100 mg/kg group were insignificant from those of vehicle control group.

Regarding beclin 1, both DR + Hesp 50 or 100 mg/kg groups showed significantly higher levels versus DR control group. Also, a significant increase in beclin 1 level in DR + Hesp 100 mg/kg group was observed versus DR + Hesp 50 mg/kg group (Figure 5F).

### 3.3. Hesperetin Suppresses mRNA Expression of the Different Markers

**Figure 6:**
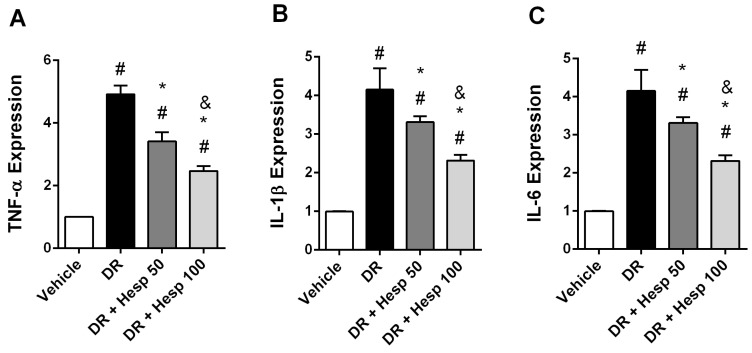

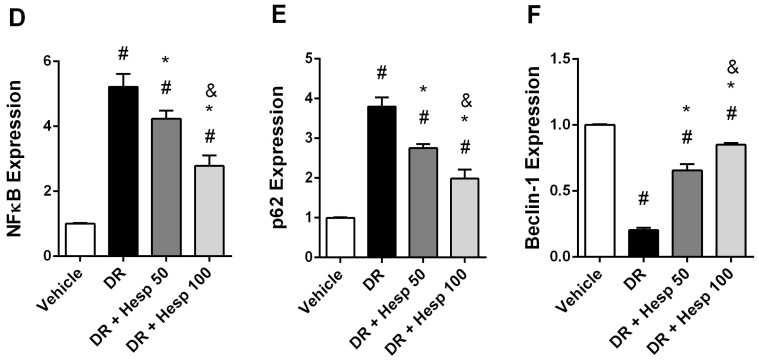
RT-PCR for the inflammation and autophagy markers in diabetic retinas. (**A**) TNF-α, (**B**) IL-1β, (**C**) IL-6, (**D**) NFκB, (**E**) beclin 1 and (**F**) p62. # Versus vehicle group, * versus DR group and ^&^ versus DR + Hesp 50 mg/kg (*p* < 0.05).

The DR control rats revealed significant fold increments in mRNA expression of the inflammation markers (TNF-α, IL-1β, IL-6, NFκB) and p62, but decreased expression of beclin 1 (Figure 6A–F). DR + Hesp 50 or 100 mg/kg groups showed significant declines in TNF-α, IL-1β, IL-6, NFκB and p62 expression. Furthermore, the DR + Hesp 100 mg/kg group showed significant declines in the expression of the same markers versus the DR + Hesp 50 mg/kg group (Figure 6A–E).

Both DR + Hesp 50 and 100 mg/kg groups exhibited significantly enhanced expression of the autophagy marker beclin 1 versus the DR control group (Figure 6F). In addition, beclin 1 expression in the DR + Hesp 100 mg/kg group was significantly higher than that observed in the DR + Hesp 50 mg/kg group. 

### 3.4. The Impact of Hesperetin on Retinal Autophagy Markers

**Figure 7:**
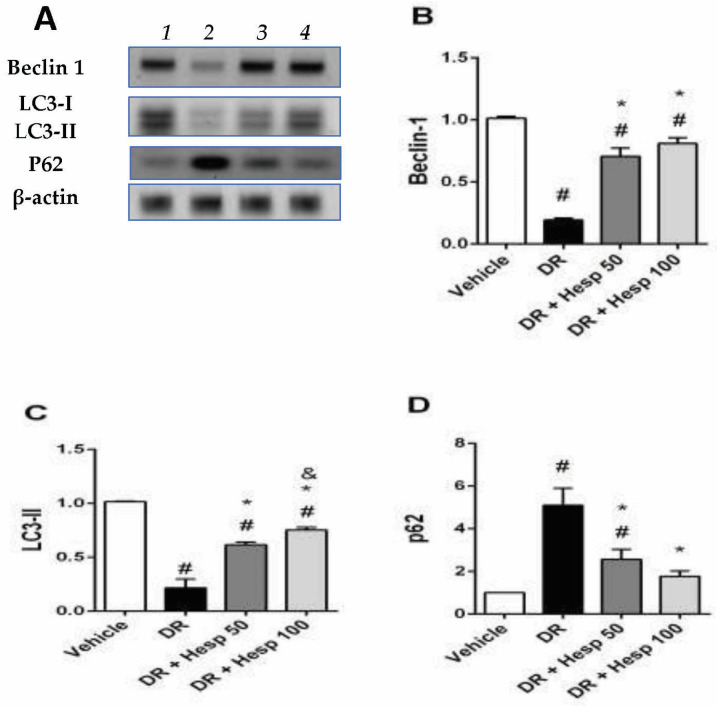
Western blot analysis for beclin 1, LC3-II and p62 proteins in retinas. (**A**) Western blot gels for samples from (1) vehicle group, (2) DR group, (3) DR + Hesp 50 mg/kg group, and (4) DR + Hesp 100 mg/kg group. (**B**–**D**) Columns represent the densities of protein bands compared to β-actin. The data are means ± SD. # Versus vehicle group, * Versus DR group, ^&^ V and versus the DR + Hesp 50 mg/kg group (*p* < 0.05).

Figure 7A shows Western blot gels for the selected autophagy markers and analysis of these bands highlighted declines in retinal beclin 1 level and LC3-II level in the DR group versus the vehicle group (Figure 7B,C). DR + Hesp 50 or 100 mg/kg showed enhanced beclin 1 and LC3-II levels versus the DR control group. Conversely, p62 proteins showed increment in the DR group, but were dose-dependently downregulated by hesperetin doses (Figure 7D).

### 3.5. Retinal Beclin 1 Immunostaining

**Figure 8:**
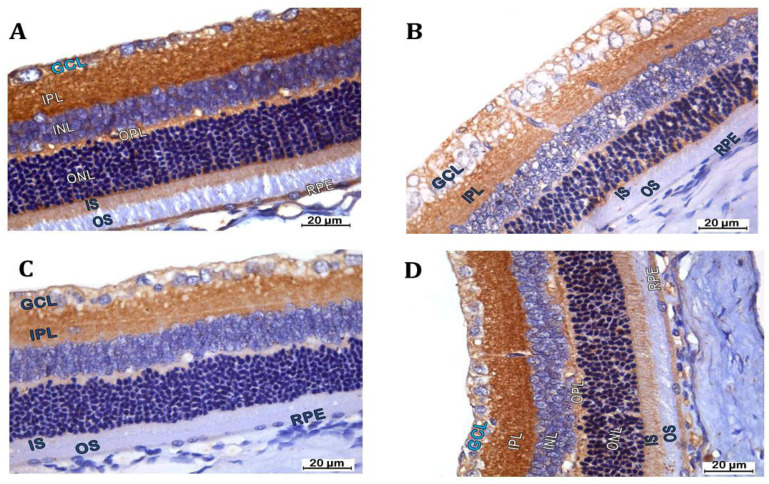

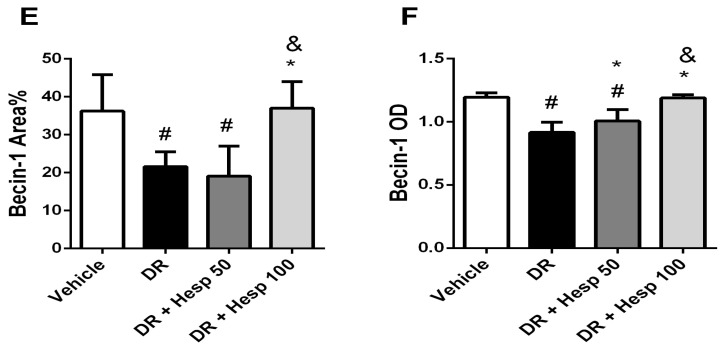
Photomicrographs of sections of the retina in different groups (beclin 1 immunostaining x630). (**A**) The vehicle group shows positive reaction for beclin 1 immunostaining in the RPE, inner segments (ISs) of rods and cones, outer plexiform layer (OPL), inner plexiform layer (IPL), GCL and NFL. The reaction appears as dark-brown cytoplasmic staining. (**B**) The DR group shows negative reaction for beclin 1 immunostaining in the RPE, and inner segments (ISs) of rods and cones. There is decreased reaction for beclin 1 immunostaining in the OPL, IPL, GCL and NFL compared to the vehicle group. (**C**) The DR + Hesp 50 group shows negative reaction for beclin 1 immunostaining in the RPE, and inner segments (ISs) of rods and cones similar to DR group. There is decreased reaction of beclin 1 immunostaining in the OPL, IPL, GCL and NFL. (**D**) The DR + Hesp 100 group shows beclin 1 reaction in the retinal layers similar to the vehicle group. (**E**) The mean color area percentage of beclin 1 immunostaining in the retinal layers. (**F**) The mean optical density (OD) of beclin 1 immunoreactivity in retinal layers. # versus the vehicle group, * versus the DR group, and ^&^ versus the DR + Hesp 50 group (*p* < 0.05).

The vehicle group showed positive reactions for beclin 1 immunostaining in the retinal pigmented epithelium (RPE), inner segments (ISs) of rods and cones, outer plexiform layer (OPL), inner plexiform layer (IPL), GCL and NFL. The reaction appeared as dark-brown cytoplasmic staining [Figure 8A]. The mean area percentage of beclin 1 immunostaining in the retinal layers was (36.19 ± 9.64), and the mean optical density (OD) of beclin 1 immunoreactivity was (1.19 ± 0.03) (Figure 8E,F). The DR group showed negative reactions for beclin 1 immunostaining in the RPE and inner segments (ISs) of rods and cones. There was decreased reaction for beclin 1 immunostaining in the OPL, IPL, GCL and NFL versus the vehicle group (Figure 8B). There was a significant decline in mean area percentage of beclin 1 immunostaining and mean optical density (OD) of beclin 1 immunoreactivity in the retinal layers compared to the vehicle group (Figure 8E,F). The DR Hesp 50 group showed negative reactions for beclin 1 immunostaining in the RPE, and inner segments (ISs) of rods and cones were similar to DR group. There was a decreased reaction of beclin 1 immunostaining in the OPL, IPL, GCL and NFL compared to the vehicle group (Figure 8C,E,F). The DR + Hesp 100 group showed beclin 1 reaction in the retinal layers similar to vehicle group (Figure 8D–F).

### 3.6. Hesperetin Improved the Retinal Histopathology Picture

#### 3.6.1. Staining with Periodic Acid–Schiff (PAS)

**Figure 9:**
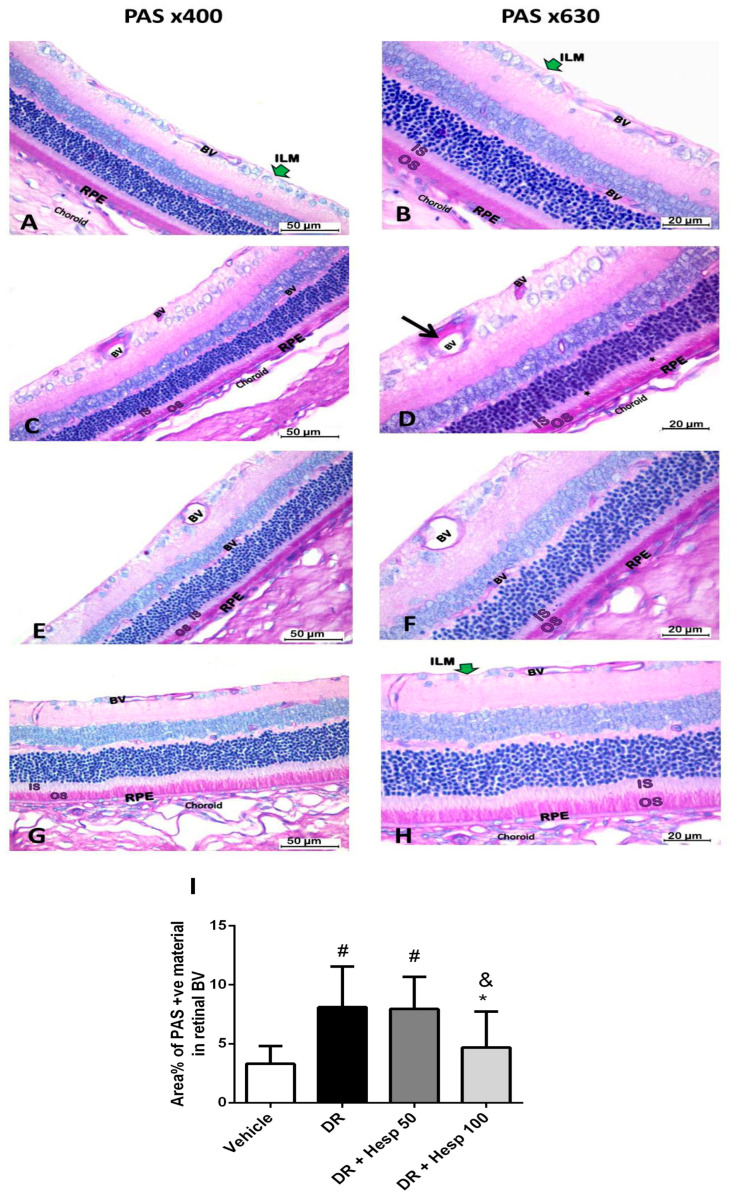
Photomicrographs of sections of the retina in different groups (PAS x400, PASx630). (**A**,**B**) The vehicle group shows PAS-positive material in the outer segment (OS) of rods and cones, basement membrane of blood vessels (BVs), inner limiting membrane (ILM), and basal lamina of RPE. (**C**,**D**) The DR group shows increased deposition of PAS-positive material in the basement membrane (black arrow) of BVs in the NFL and GCL. The ILM appears normal. There are vacuoles (star) in the inner segment (IS) of rods and cones. (**E**,**F**) The DR + Hesp 50 group shows decreased PAS-positive area in the wall of BVs compared to the DR group. (**G**,**H**) The DR + Hesp 100 group shows normal PAS reaction in the retinal layers similar to the vehicle group (**I**) Column chart presents the mean percentage of PAS-positive area in the wall of retinal BVs among different groups. [#] Versus vehicle group, [*] versus DR group, and [&] versus DR + Hesp 50 group (*p* < 0.05).

Periodic acid–Schiff (PAS) staining was used to evaluate the thickening of basement membrane of retinal BV in different groups. Thickening of capillary basement membrane is an early histological change in DR. It is seen in the early stage of non-proliferative diabetic retinopathy (NPDR). The vehicle group demonstrated PAS-positive reactions in the outer segment (OS) of rods and cones, inner limiting membrane (ILM), basement membrane of blood vessels (BVs), and RPE basal lamina (Figure 9A,B). The color area percentage of PAS-positive reactions in the wall of BVs was 3.3 ± 1.51 (Figure 9I). The DR group showed deposition of PAS-positive material in the basement membrane of BVs of the GCL and NFL (Figure 9C,D). We observed a significant increment in area percentage of PAS-positive reactions in the wall of BVs compared to vehicle group (Figure 9I). The DR + Hesp 50 group showed decreased PAS-positive material in the wall of BVs of the GCL and NFL compared to the DR group (Figure 9E,F), but the average area percentage of PAS-positive material in the wall of retinal BVs was greater than that recorded in the vehicle group (Figure 9I). The DR + Hesp 100 group showed normal PAS reaction similar to the vehicle group (Figure 9G,H). There was a significant decline in the area percentage of PAS-positive reaction in the wall of BVs in comparison to the DR group (Figure 9I).

#### 3.6.2. Histology Staining with Routine Hematoxylin and Eosin

**Figure 10:**
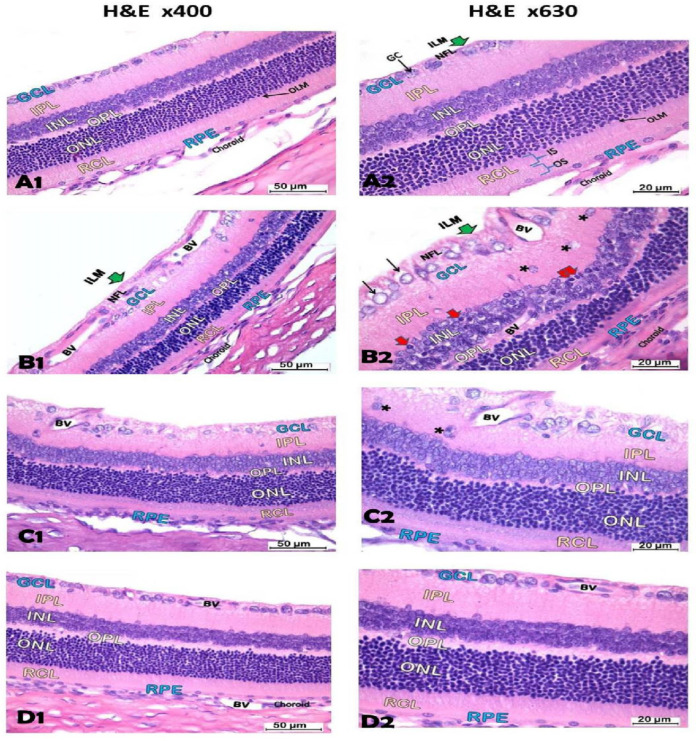
Photomicrographs of sections of the retina in the experimental groups. (**A1**,**A2**) The vehicle group shows normal structure of the retina with 10 layers, the retinal pigmented epithelium (RPE) has a single row of cells with oval nuclei, the layer of rods and cones (RCL) has outer (OS) and inner (IS) segments, outer limiting membrane (OLM), outer nuclear layer (ONL) with rods and cones appearing with dark nuclei, pink appearance for the outer plexiform layer (OPL), pale stained nuclei in the inner nuclear layer (INL), inner plexiform layer (IPL), the ganglion cell layer (GCL) contains large ganglion nerve cells (GC) with vesicular nuclei, optic nerve fiber layer (NFL) and inner limiting membrane (ILM). The choroid (C) overlies the retina. (**B1**) The DR group shows decreased thickness of RCL, ONL, and INL compared to vehicle group. The RPE layer is thinned with flattened nuclei. Dilated blood vessels (BVs) with thick walls are seen in the GCL and NFL. (**B2**) Higher magnification image of DR group shows the INL with many pyknotic dark nuclei (red arrows). Some nuclei are displaced in the IPL (asterisks). GCL shows swollen vacuolated GC (black arrows) with nuclei that have chromatin margination. Blood vessels (BVs) can be seen in the OPL and INL, and dilated BVs are apparent in the GCL and NFL. (**C1**,**C2**) The DR + Hesp 50 group shows increased thickness of the retinal layers, RCL, ONL and INL compared to DR group. RPE has row of cells with oval nuclei. Nuclei of ONL and INL appear normal. Some nuclei are displaced in the IPL (star). Dilated BVs are seen in the GCL and NFL. (**D1**,**D2**) The DR + Hesp 100 group shows normal structure of the retina similar to vehicle group [H&E x 400, H&E x 630].

Pictures from the vehicle group demonstrated regular structure of the retina with 10 layers, retinal pigmented epithelium (RPE), RCL, outer limiting membrane (OLM), ONL with dark nuclei of rods and cones, outer plexiform layer (OPL) with pink appearance, INL with pale stained nuclei, inner plexiform layer (IPL), the GCL contained large ganglion nerve cells (GC) with vesicular nuclei, optic nerve fiber layer (NFL) and inner limiting membrane (ILM) (Figure 10A1,A2). The DR group showed many pyknotic nuclei in the INL, while the GCL showed swollen vacuolated GC with nuclei that had chromatin margination. Dilated blood vessels with thick walls were present in the GCL and NFL (Figure 10B1,B2). The DR + Hesp 50 group showed a decline in the thickness of the RCL, ONL, and INL compared to the vehicle group. Dilated BVs were apparent in the NFL and GCL (Figure 10C1,C2). The DR + Hesp 100 group showed improvement in the structure of the retina, which appeared nearly normal (Figure 10D1,D2).

The histological findings in the DR group included decreased thickness of the RCL, ONL, and INL, pyknotic nerve cells in the INL, and thickened dilated BVs in the GCL and NFL, which are correlated with early-stage DR (non-proliferative diabetic retinopathy, NPDR) [45]. Increased thickness of the NFL and GCL in our study is associated with edema and leakage of thickened capillaries in the NFL which is also an early histological change with DR [45]. In HE-stained sections, we could not observe new vessel formation (neovascularization) at the interface between the retina and vitreous or vitreous hemorrhage that characterizes the severe stage of DR (proliferative DR, PDR) [46,47], which may be due to the short duration of the experiment.

**Figure 11:**
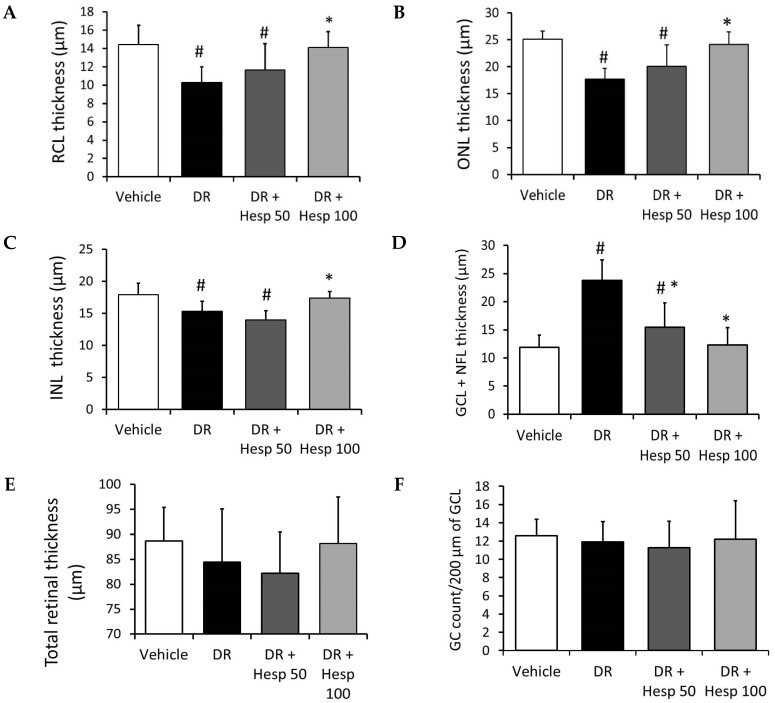
Comparison of retinal layer thickness among different groups. (**A**) RCL thickness, (**B**) ONL thickness, (**C**) INL thickness, (**D**) GCL + NFL thickness, (**E**) total retinal thickness (all measured in µm), and (**F**) count of GC/200 µm length of GCL. [#] versus vehicle group and [*] versus DR group.

The total thickness of retinas was 88.69 µm ± 6.73. The RCL thickness was 14.42 µm ± 2.09, ONL thickness 25.15 µm ± 1.5, INL thickness 17.9 µm ± 1.8, and GCL and NFL thickness 11.9 µm ± 2.18. The mean count of GC/200 µm length of GCL was 12.6 ± 1.8 (Figure 11A–F). The DR group displayed a significant decline in the thickness of the RCL, ONL, and INL compared to the vehicle group, while the GCL and NFL exhibited an increment in comparison to the vehicle group (Figure 11A–F). The DR + Hesp 50 group showed significant declines in the thickness of the RCL, ONL, and INL versus the vehicle group, while the GCL and NFL showed significant (*p* < 0.05) improvement compared to the DR group (Figure 11A–F). The DR + Hesp 100 group demonstrated significant improvements in the thickness of the retinal layer measurement (RCL, ONL, INL and GCL and NFL) compared to the DR group (Figure 11A–F). There was no significant difference in the count of GC/200 µm length of GCL among the study groups (Figure 11F).

## 4. Discussion

Diabetic retinopathy poses a serious risk to the vision of older people who have diabetes. Despite extensive research and the demonstrated efficacy of standard DR treatments, DR continues to be dangerous. DR is a multifactorial illness, and numerous studies have shown that a number of risk factors, including hyperglycemia, hypertension, dyslipidemia, prothrombotic state, oxidative stress, proinflammatory cytokines, angiogenesis stimulatory molecules, and others, are linked to the disease [48,49]. Tight glycemic control is the current gold standard for DR prevention [50]. Nevertheless, extremely strict glycemic management (HbA1c of 6% or less) is not advised because it may raise the risk of hypoglycemia-related cardiovascular events and mortality [51]. Hence, further treatment options are strongly warranted.

Hesperetin is an inexpensive flavanone with strong antioxidant activity that is mainly obtained from citrus species [52]. It is also known to have anti-inflammatory and COX2-inhibitory action [53], neuroprotective activity [54] and to decrease vascular permeability [55]. Hence, the current study intended to provide a promising strategy through modulation of autophagy by hesperetin for the management of experimental DR.

In the current computational approach, the targeted gene/protein investigation revealed a list of 446, 589 and 344 genes for neurodegeneration pathways, DR and autophagy. Of them, 115 of intercalating genes and proteins shared between the three pathways indicated a rationale for studying them. Furthermore, KEGG enrichment analysis explored the enriched pathways in the three processes. Interestingly, the analysis revealed that most of the genes were enriched in pathways, as autophagy and neurodegeneration and the top pathways were interconnected and intercommunicating. This provided a strong rationals for studying these connections in the experimental study. PPI analysis in STRING also revealed an undeniable interaction between the genes and proteins. Beclin 1, LC3-II, NFκB, TNF-α, IL1β and IL6 were chosen as representatives from the two clusters, and they all maintained a strong functional protein association. Hence, we selected these markers to be measured in the experimental study.

In our experimental study, the protein levels of IL-1β, IL-6, NFκB, and TNF-α were elevated in rats with DR. In the presence of hyperglycemia, the proinflammatory cytokines (TNF-α, growth factors, IL-1, IL-6) increase and are believed to be involved in the initiation and exacerbation of inflammation in DR. Furthermore, chronic inflammation generates fibrotic processes, which in turn induce scar formation and ultimately retinal separation [56,57]. It is known that hyperglycemic stress activates microglia in the retina, which in turn increases oxidative stress and induces proinflammatory cytokines like IL-1β, IL-6, and TNF-α, as well as chemokines and adhesion molecules, resulting in an increment in vascular permeability, pericyte loss, and the appearance of microaneurysms [58]. Regarding NFκB, there was elevation in its mRNA expression and the total amount of NFκB protein in the retinal homogenate; however, previous studies showed that the phosphorylated form (p-NFκB) was elevated in hyperglycemia models [59,60]. This may be attributed to the fact that in our study, the total measured NFκB included both phosphorylated and non-phosphorylated forms. If the p-NFκB were measured, we might have found elevated levels also. Differences in hyperglycemia models (in vitro and in vivo) in addition to the differences in the duration of diabetes may influence these points.

Additionally, elevated concentrations of inflammatory cytokines in retinal tissue homogenates have been observed in diabetic rats. On the contrary, hesperetin attenuated DR in rats through downregulation of IL-1β, IL-6, NFκB, and TNF-α in the retinal tissue homogenates, which confirmed the inhibitory effects of hesperetin on inflammatory burdens and matches previous evidence that illustrated hesperetin ameliorated the inflammation in diabetic nephropathy in rats [61].

It is generally accepted that abnormalities in autophagy have a role in the development of diabetes. According to previous evidence, high-glucose conditions stimulate mitophagy, a subtype of macroautophagy that selectively eliminates damaged mitochondria [62]. DR has evolved different complicated mechanisms to deal with environmental stress as a result of several molecular interactions [63]. In response to various environmental stress, autophagy is frequently triggered to preserve normal cellular function. Recent studies verified that autophagy is involved in development of DR, where high blood glucose led to the activation of such inflammatory mediators as inflammasomes and IL-1β, and accordingly autophagy inhibition [64,65]. Autophagy is inhibited in DR by lysosome membrane permeabilization, which decreases the degradative capacity of autophagy through activation of inflammatory mediators [66]. Amato et al. reported that autophagy could be suggested as a molecular target for neuroprotective treatment strategies aiming to alleviate DR [67].

Kong et al. evaluated autophagy in diabetic mice and found that there was an upregulation of autophagy in mice with diabetes, as evidenced by increases in autophagy markers LC3-II and beclin 1, along with reduction in p62 [68]. It is crucial to note that autophagy has contradictory effects in DR, increasing cell survival in low-stress conditions, but leading to programmed cell death in the presence of high-stress stimuli [13]. As an autophagy-specific marker during induction of autophagy, light chain 3 (LC3) protein is coupled with phosphatidylethanolamine and localized to autophagic membranes [69]. For phagophore elongation and autophagosome closure, LC3 complexes function as crucial ubiquitin-like conjugating mechanisms [70].

Beclin 1 is a substantially conserved eukaryotic protein, regulates vesicle transfer, and excites autophagy [71]. Beclin 1 plays a crucial function in facilitating the formation of autophagosomes through its interaction with Vps34. The maturation of autophagosomes, the extrusion of lipid membranes, and the accumulation of cellular cargo can all be accelerated by the beclin 1–Vps34 complex [72]. P62 functions as a receptor for cellular cargo, securing it to the autophagosome membrane before its degradation in the lysosome. Decreased p62 primarily indicates increased autophagy [73].

We confirmed in the present study that p62 was upregulated in the DR group, whereas beclin 1 and LC3-II were downregulated, showing that autophagy was suppressed in DR rats when exposed to high glucose levels, but beclin 1 and LC3-II were elevated after hesperetin administration, indicating the induction of autophagy, in accordance with a recent study that reported hesperetin-induced autophagy may protect cells against apoptosis by acting as an adaptive response in human leukemia cells [74]. Therefore, hesperetin has promise as a supplement to treatment for DR.

Previous studies match our results. In STZ-induced diabetic mice, Mao and colleagues found that miR-204-5p suppressed the expression of LC3B-II, hence inhibiting autophagy in DR [75]. Luo et al. found that in db/db mice, p62 was significantly upregulated, while pro-autophagy proteins like beclin 1, LC3-II, and Atg5 were significantly downregulated [76]. In the other hand, upregulation of beclin 1 and enhanced immunoreactivity for LC3-II in the OPL were found in rats with diabetes caused by STZ [77] which may be attributed to the regulation of autophagy in diabetic animals and may differ based on disease development and time periods studied. Fu and coworkers (2020) found that LC3-II protein levels in retinal ganglion cells of C57BL/KsJ-db/db mice, an experimental spontaneous diabetes model, varied with age without a discernible pattern [78]. Different pharmacological strategies have been tested to manage dysfunctional autophagy as a promising modality to overcome neurodegenerative disorders [79]. Promising results have been demonstrated with a natural isoflavone, biochanin A. This natural agent restored autophagy and protected against neurodegeneration [79,80,81]. 

Hesperetin is a bioflavonoid with anti-inflammatory, anti-oxidative, and anti-diabetic properties [82,83]. In the current results, hesperetin protected against the pathological findings recorded in the retinas of STZ-diabetic rats: it downregulated the inflammatory burden (IL-1β, IL-6, NFκB, and TNF-α) and enhanced autophagy (elevated beclin 1 and LC3B-II, but decreased p62). Similarly to our findings, previous studies showed that hesperetin can prevent cataracts and presbyopia in animals [84,85]. Hesperetin has three hydroxyl groups that possess great antioxidant capacity and are able to activate cellular antioxidant enzymes [86]. Recently, Lai et al. studied the ability of hesperetin to overcome oxidative stress and examined the mechanisms by which it may protect against AGE-induced neuronal cellular damage. They documented that hesperetin was able to upregulate the antioxidative mechanisms contributing to human SH-SY5Y neuroblastoma cells against AGE-induced ROS [87]. Similarly, Doki et al. found that therapeutic doses of hesperetin attenuated glycation of lens proteins and AGE generation [52]. Furthermore, Kara et al. reported that hesperetin provided protection from apoptosis in a rat ischemia–reperfusion retinal injury model [88]. In another study, the same group studied the ability of hesperetin to rescue diabetes induced neurovascular complications in rats in addition to combating retinal oxidative stress, neuroinflammation and apoptosis [40].

Shimouchi et al. documented that hesperetin protected ganglion cells from I/R injury in mice via the modulation of cell death signaling in addition to providing anti-inflammatory action through reduction of the upregulated expression of IL-1β from activated microglia [89]. Also, Hwang et al. reported neuroprotective action provided by the citrus flavanones in face of cytotoxicity induced by H2O2 in PC12 cells [90]. Likewise, Kumar et al. reported that hesperetin amends retinal vasculopathy induced via hyperglycemia in experimentally diabetic rats [91].

A naturally occurring flavonoid, hesperetin possesses a number of pharmacological characteristics, such as anti-inflammatory and antioxidative capabilities. Several chronic inflammatory diseases have been treated and countered with hesperetin [23,92]. Hesperetin has also been demonstrated to strongly prevent proinflammatory cytokines (TNF-α, IL-1β, and IL-6) [93]. In addition, hesperetin has been shown to reduce inflammation in a variety of cell types by regulating the p38 mitogen-activated protein kinase (MAPK) signaling pathway and raising the amounts of antioxidant proteins in mouse brains, such as nuclear factor erythroid 2-related factor 2 (Nrf2) [23]. By raising catalase, superoxide dismutase, and glutathione (GSH) ratios, hesperetin significantly decreased lipid peroxidation levels and protein oxidation while enhancing the expression of antioxidant defense. When STZ-induced type 1 diabetes was tested, hesperetin demonstrated a significant increase in GSH levels, as well as the antioxidant genes Nrf2 and heme oxygenase 1 [94]. It has been reported that in Neuro-2A cells, hesperetin impairs insulin-stimulated glucose uptake and A1–42-induced autophagy. The results of this study indicate that by regulating autophagy, hesperetin may be a viable therapeutic agent to stop the progression of neurodegeneration [95]. In numerous experimental models of neurodegenerative diseases, hesperetin has been shown to have neuroprotective effects. Through the PI3K-Akt and MAPK pathways, as well as the recruitment of neuronal progenitor cells that influence astrocytes, this flavonoid enhances neuronal survival [26,96]. 

## 5. Conclusions

In conclusion, the current results demonstrated defective autophagy in experimental DR as indicated by increased p62 and defective beclin 1 and LC3-II levels and highlighted that hesperetin improved autophagy and protected against retinal pathological changes observed in rats. Hence, we can suggest adding hesperetin in the form of citrus species in the diet of DR patients. Additional investigation into defective autophagy at different stages of DR with the effective clinical implementation of downstream autophagy modulation presents promising opportunities for the development of therapies in DR.

## Figures and Tables

**Table 1:** Primer sequence for genes tested in the retinal samples.

Target Gene	Primer Sequence	Product Size	ReqSeq
*TNF-α*	Forward primer: GCAGATGGGCTGTACCTTATC Reverse primer: GGCTGACTTTCTCCTGGTATG	121 bp	NM_012675.3
*IL-6*	Forward primer: TTCAACATGGCAGACGACGA Reverse primer: TGCTCTAGTATTTGAAGGTATGGG	146 bp	NM_001276711.2
*IL-1*	Forward primer: TCCTCTGTGACTCGTGGGAT Reverse primer: GTTTGGGATCCACACTCTCCA	309 bp	NM_031512.2
*NFκB*	Forward primer: GCCAGAGTCATTCAGAGCAATA Reverse primer: GTTGGATGGTCTTGGTCCTTAG	160 bp	NM_012589.2
*Beclin 1*	Forward primer: TGGCACAGCGGACAATTTGG Reverse primer: AACAGTACAACGGCAACTCCTTA	232 bp	NM_001034117.1
*P62*	Forward primer: CAGCTGCTGTCCGTAGAAATTG Reverse primer: ACCCGCTCTTTCAGCTTCAT	113 bp	NM_130405.2
*GAPDH*	Forward primer: AGTTCAACGGCACAGTCAAG Reverse primer: TACTCAGCACCAGCATCACC	119 bp	NM_017008.4

## Data Availability

Data are available from the authors upon request.

## Supplementary Materials

The following supporting information can be downloaded at: https://www.mdpi.com/article/10.3390/biomedicines12030552/s1, Figure S1: KMEANS clustering algorithm analysis of protein interaction network in the query gene list showing them arranged in two major clusters. The dotted lines represent edges connecting the two clusters. Analysis was undertaken using STRING v.12.0.
