# Supplementary material for: Computational and Experimental Approaches Exploring the Role of Hesperetin in Improving Autophagy in Rat Diabetic Retinopathy
Source: Biomedicines. 2024 Mar 1;12(3):552. doi: 10.3390/biomedicines12030552 (PMC10968489; doi:10.3390/biomedicines12030552)
Supplement: Supplementary file 1 [file biomedicines-2833034-supplementary.pdf › biomedicines-2833034-supplementary.pdf]

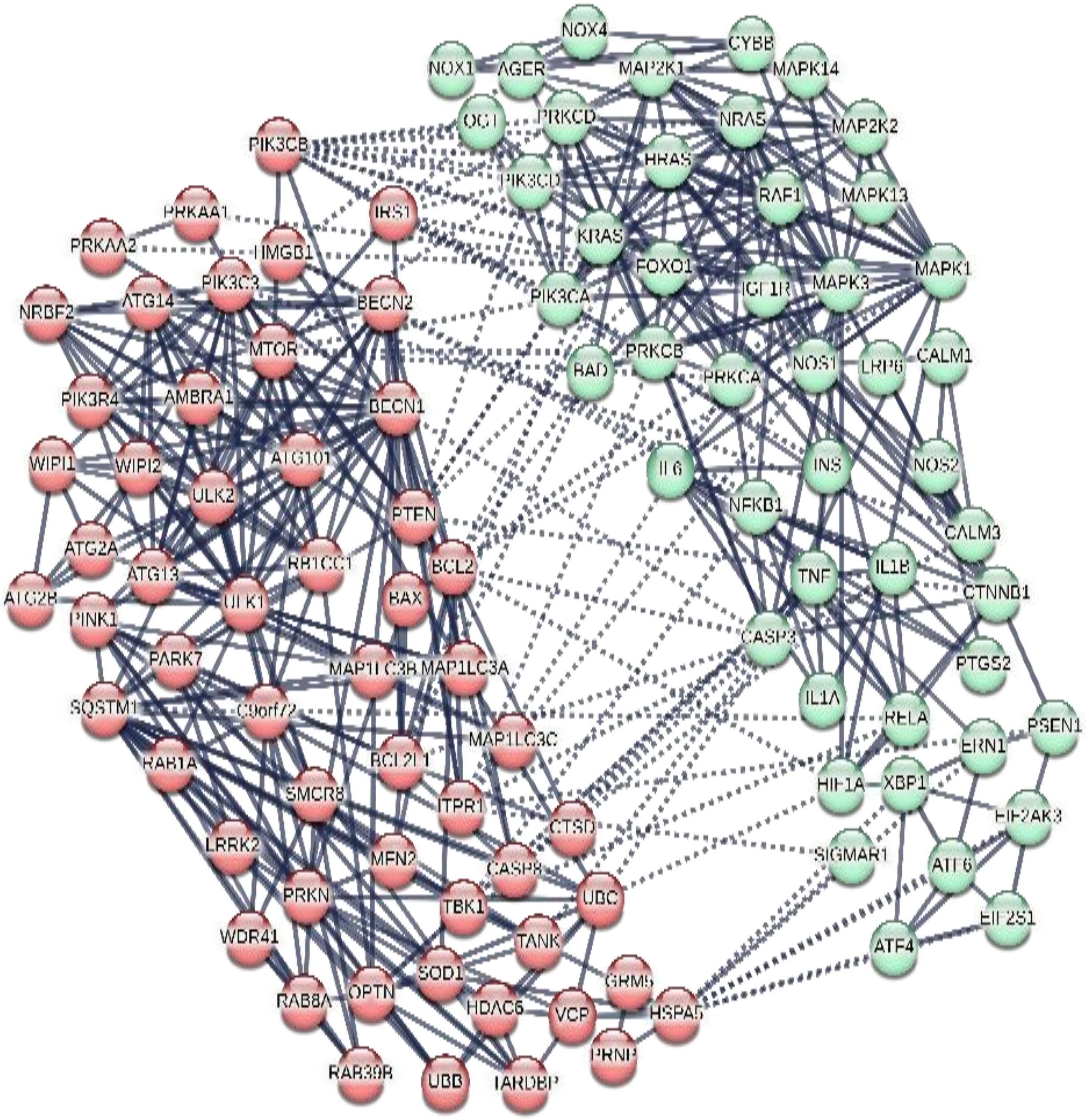

Supp. Figure S1. KMEANS clustering algorithm analysis of protein interaction network to the query gene list showing them arranged in 2 major clusters. The dotted lines represent edges connecting the two clusters. Analysis was done using STRING v.12.0.
